# Supplementary material for: Spin-Orbit induced phase-shift in Bi2Se3 Josephson junctions
Source: Nat Commun. 2019 Jan 10;10:126. doi: 10.1038/s41467-018-08022-y (PMC6328588; doi:10.1038/s41467-018-08022-y)
Supplement: Supplementary file 1 — Supplementary Information [file 41467_2018_8022_MOESM1_ESM.pdf]

Spin-Orbit induced phase-shift in Bi_2Se_3 Josephson junctions

A. Assouline et al.

Supplementary Figures

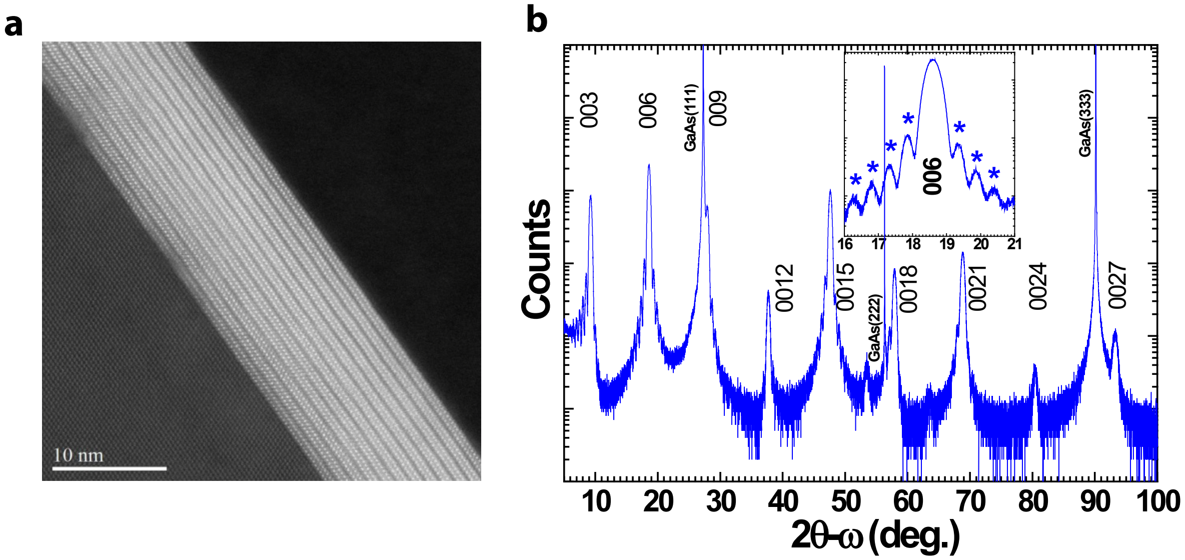

Supplementary Figure 1: **Structure of the MBE grown Bi_2Se_3 thin films. a** High-angle annular dark-field scanning transmission electron microscopy (HAADF-STEM) cross-section image of Bi_2Se_3 film with 12 quintuple layer (1 QL $\approx$ 1 nm). Each QL is delineated by the fringes with darker contrast located at the weak inter-QL bonds in van-der-Waals gap between each QL. **b** X-ray diffraction spectrum showing the crystalline structure of the films on the GaAs(111) substrate with highly directed 003-type reflections of the Bi_2Se_3 film along 111-axis of GaAs. The (006) Bragg reflection is enlarged (in insert) with Kiessig fringes (indicated by stars) which are used to determine the film thickness, about 18 QLs for this film.

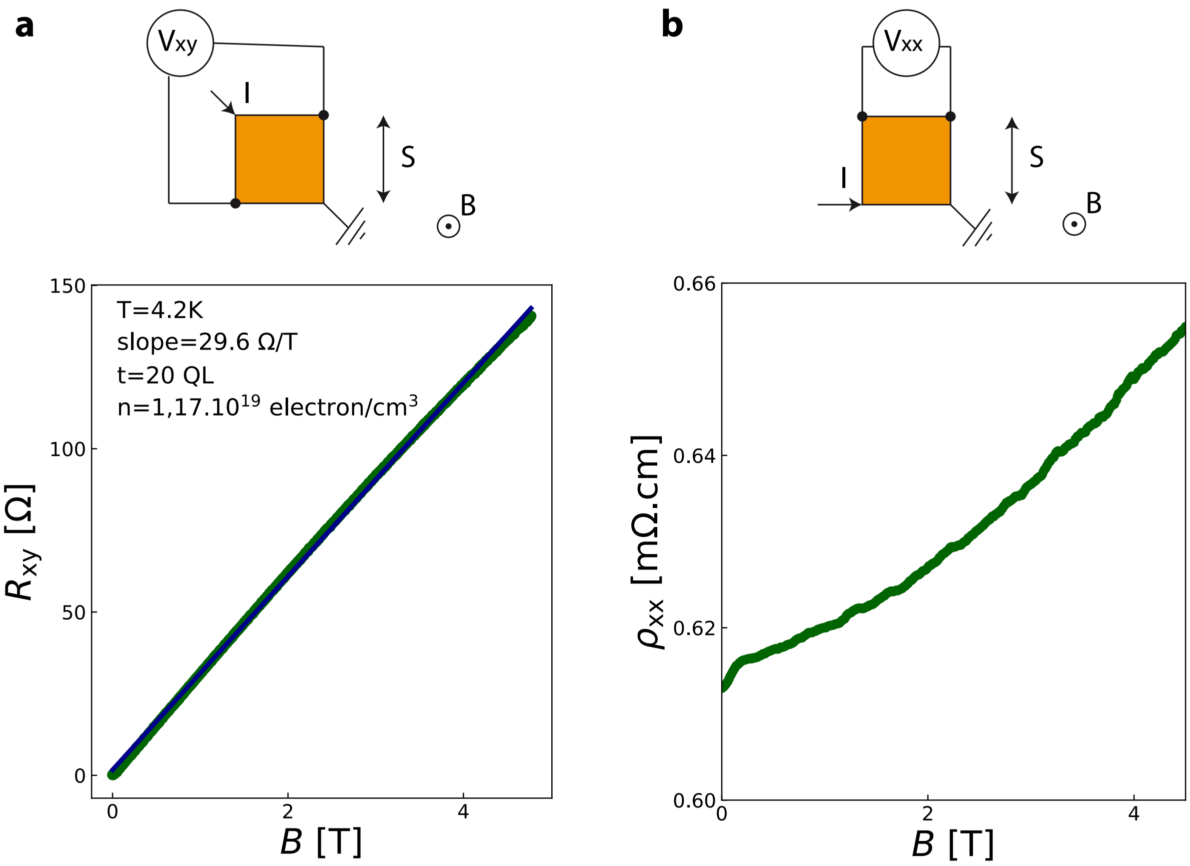

Supplementary Figure 2: **Transport properties of the Bi_2Se_3 thin films.** Top : Sketch of electrical connections used for measurements of transport properties in the Van der Pauw geometry. The measurements are realized on microfabricated squares of lateral size 380 μm. Bottom : The plots show the transverse Hall resistance $R_{\mathrm{H}} = 29.6\ \Omega.\mathrm{T}^{-1}$ (left panel) and longitudinal resistivity $\rho_{xx} = 0.61$ mΩ.cm (right panel) measured at $T = 4.2$ K. From the Hall resistance, an electronic density $n = \frac{1}{teR_{\mathrm{H}}} = 1.2\ 10^{19}$ electrons.cm^{-3} is determined. Using the effective mass $m^* = 0.25\ m_e$, Ref. [1], and the Fermi gas relation between the carrier density and the Fermi velocity, $n = \frac{(m^* v_{\mathrm{F}})^3}{3\pi^2\hbar^3}$, one finds $v_{\mathrm{F}} = 3.210^5$ m/s. From the Boltzmann relation between the resistivity and the elastic scattering time τ, $\rho_{xx} = m^*/ne^2\tau$, one finds $\tau = 0.13$ ps and the elastic mean free path $\ell = v_{\mathrm{F}}\tau = 42$ nm. Because this distance is smaller than the length of the junction $L = 150$ nm, the system is better described in the diffusive regime, where the diffusion constant is obtained from $D = \frac{v_{\mathrm{F}}\ell}{3} = 40\ \mathrm{cm}^2\mathrm{s}^{-1}$.

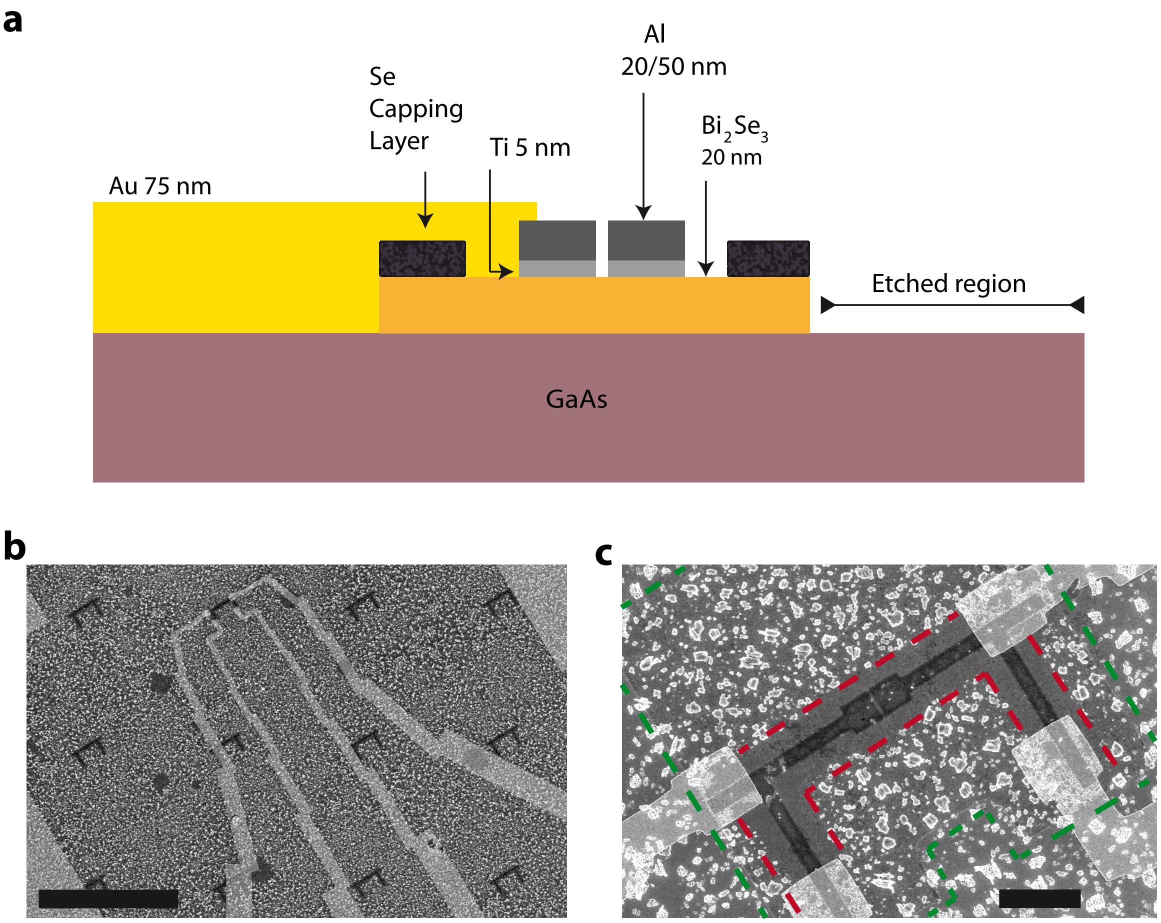

Supplementary Figure 3: **Sketch and SEM images of devices. a** Lateral view of the device. **b** Scale bar 100 μm. On these MBE grown films, many junctions are fabricated and examined by SEM lithography. The best junctions are selected and connected by Ti5 nm/Au75 nm electrodes. **c** Scale bar 10 μm. The white grains at the surface are Se grains. Before deposition of the Al electrodes (dark area), the Se capping layer is removed by chemical etching in an NMF solution of Na_2S. Because of the undercut in the PMMA resist, the area on which Se is removed extends beyond the area where the Al electrodes are evaporated. This area where Se is removed is clearly visible and indicated by dashed red lines. After evaporation of the superconducting Al electrodes, the Bi_2Se_3 film is etched to isolate the junction, the etched contour is visible and highlighted by the dashed green lines.

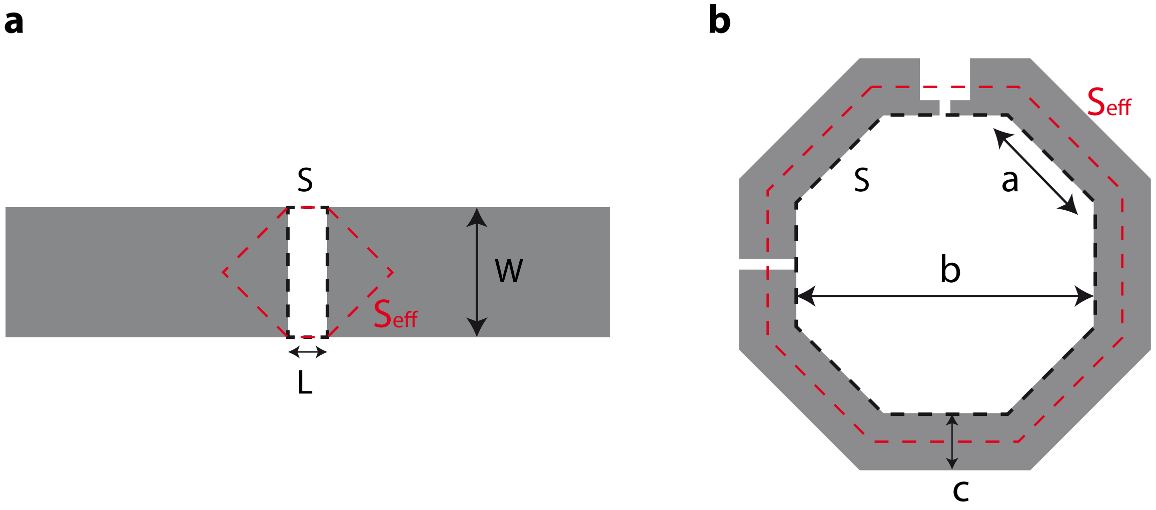

Supplementary Figure 4: **Effective surface areas due to flux focusing. a** Sketch of a junction of length L and width W. The junction area highlighted by the black dashed lines is $S = LW$. As described in the Supplementary Note 3, the flux lines present within the red dashed lines are diverted into the junction area when the electrodes become superconducting. **b** Sketch of a the Josephson interferometers used in the main text to detect the anomalous phase shift φ_0. The junction area is $S = b^2 - a^2$, where $a = 2.1$ μm and $b = 5$ μm. Due to the finite width of the electrode, $c = 1$ μm, the effective surface is $S_{eff} = (b+c)^2 - (a + c\tan(\pi/8))^2$ and can be re-written $S_{eff} = S + S_{focalized}$ with $S_{focalized} = c(2b + c - 2a\tan(\pi/8) - c^2\tan^2(\pi/8))$.

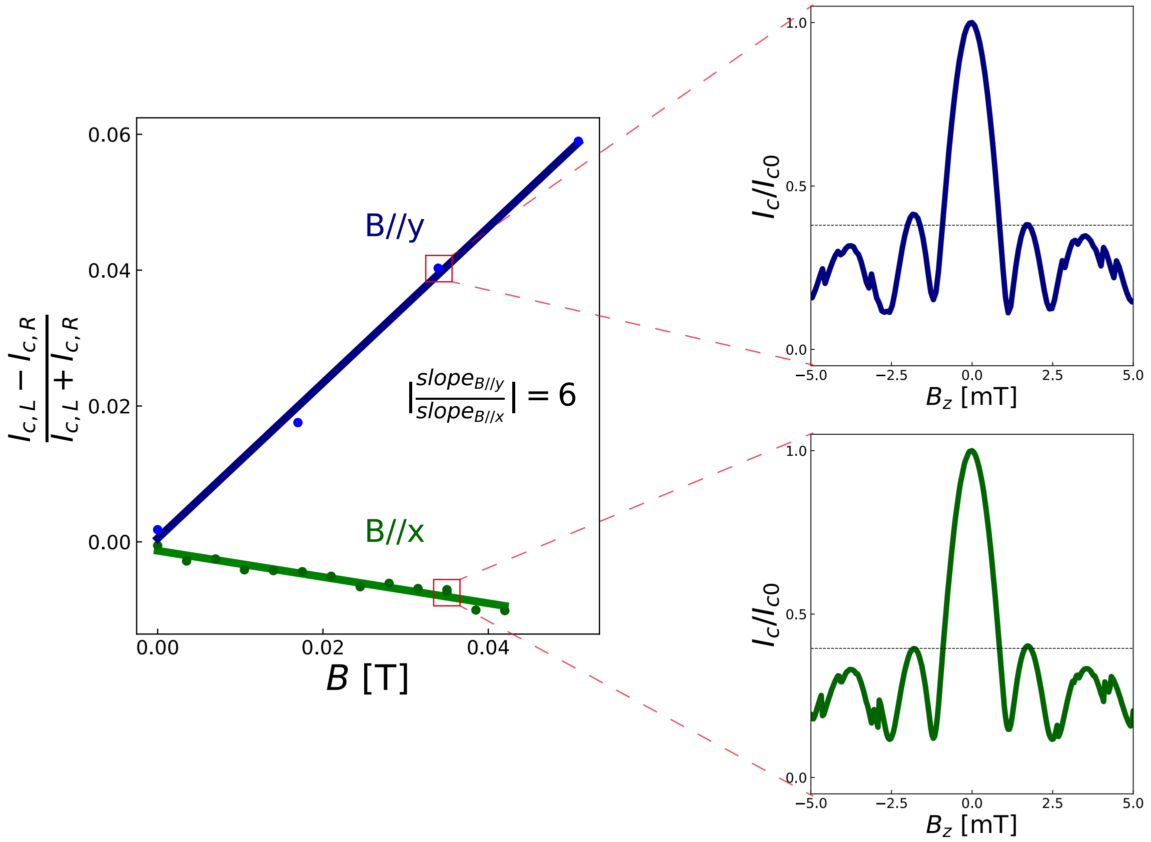

Supplementary Figure 5: **Fraunhofer pattern as a function of in plane magnetic field orientation.** To quantify the asymmetry, we show the parameter $\frac{I_{\mathrm{c,L}}-I_{\mathrm{c,R}}}{I_{\mathrm{c,L}}+I_{\mathrm{c,R}}}$ as a function of the amplitude of magnetic field, where $I_{\mathrm{c,L}}$ and $I_{\mathrm{c,R}}$ correspond to the critical current of the first lobe at negative and positive B_z, respectively. The blue and green curves correspond to in-plane magnetic fields oriented respectively perpendicular and parallel to the current direction. For both orientations, the asymmetry parameter is zero in the absence of in-plane magnetic field. For a finite amplitude of the magnetic field, the asymmetry is much more pronounced when the magnetic field is oriented in the y direction than in the x direction.

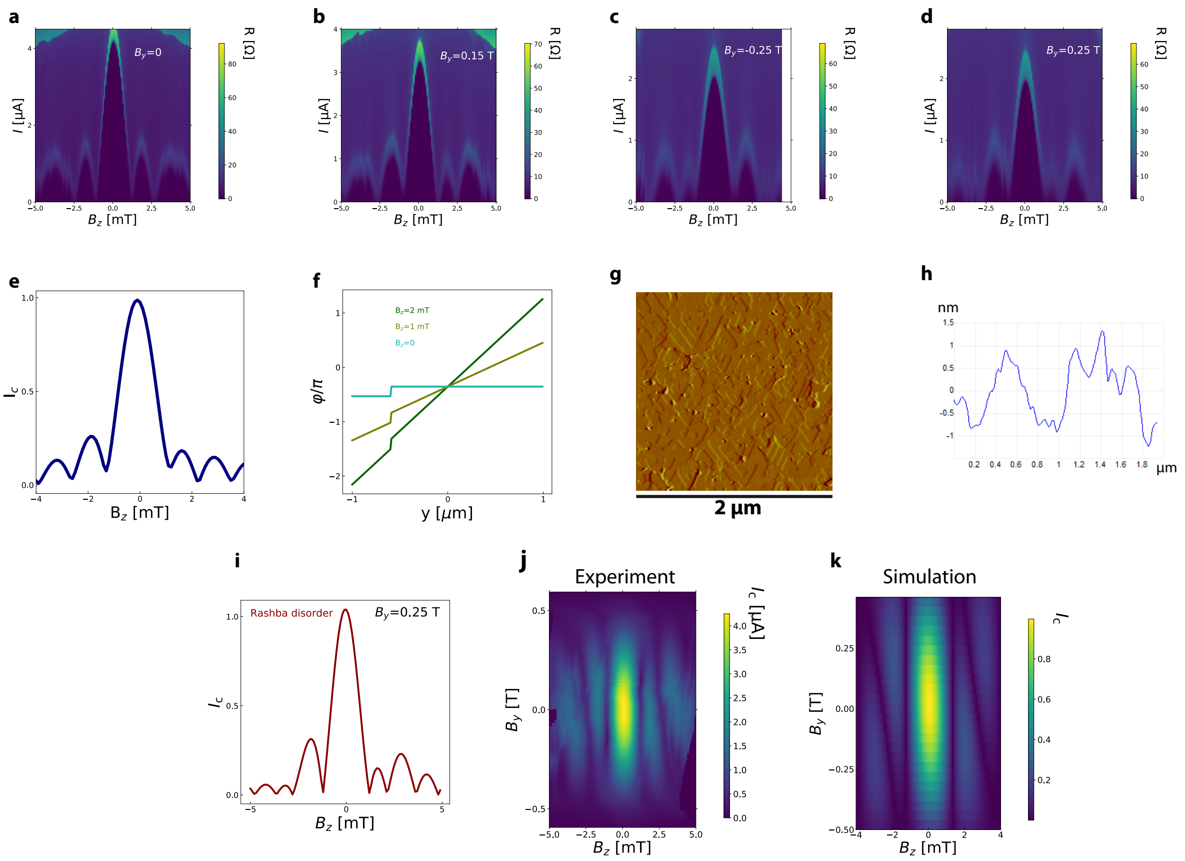

Supplementary Figure 6: **Simulation of the anomalous Fraunhofer pattern. abcd** Differential resistance maps dV/dI as a function of current and magnetic field B_z for different values of the in plane magnetic field B_y. **e** An asymmetric Fraunhofer pattern can be generated simply by a jump in the phase difference between the two superconductors along the direction y as plotted panel **f**. This phase jump can be generated by the in-plane magnetic field in presence of spin-orbit coupling. **g** 2 μm $\times$ 2 μm AFM topographic image of the thin film. **h** Typical line profile of the sample topography extracted from AFM image. The film thickness changes by $\pm$1 nm (1 QL) over an average distance of $l_{\rm d}$ $\approx$1 μm. **i** Simulation of the Fraunhofer pattern at B_y=0.25 T with a Rashba parameter which changes by 0.01 eVÅ over a length $l_{\rm d}$ $\approx$1 μm. **j** Experimental current map showing the evolution of the asymmetry with the magnetic field. **k** Simulation of the anomalous pattern using Supplementary Eq.[7] 7, where phase jumps are induced along the y direction as a consequence of the change in the Rashba coefficient α with the film thickness.

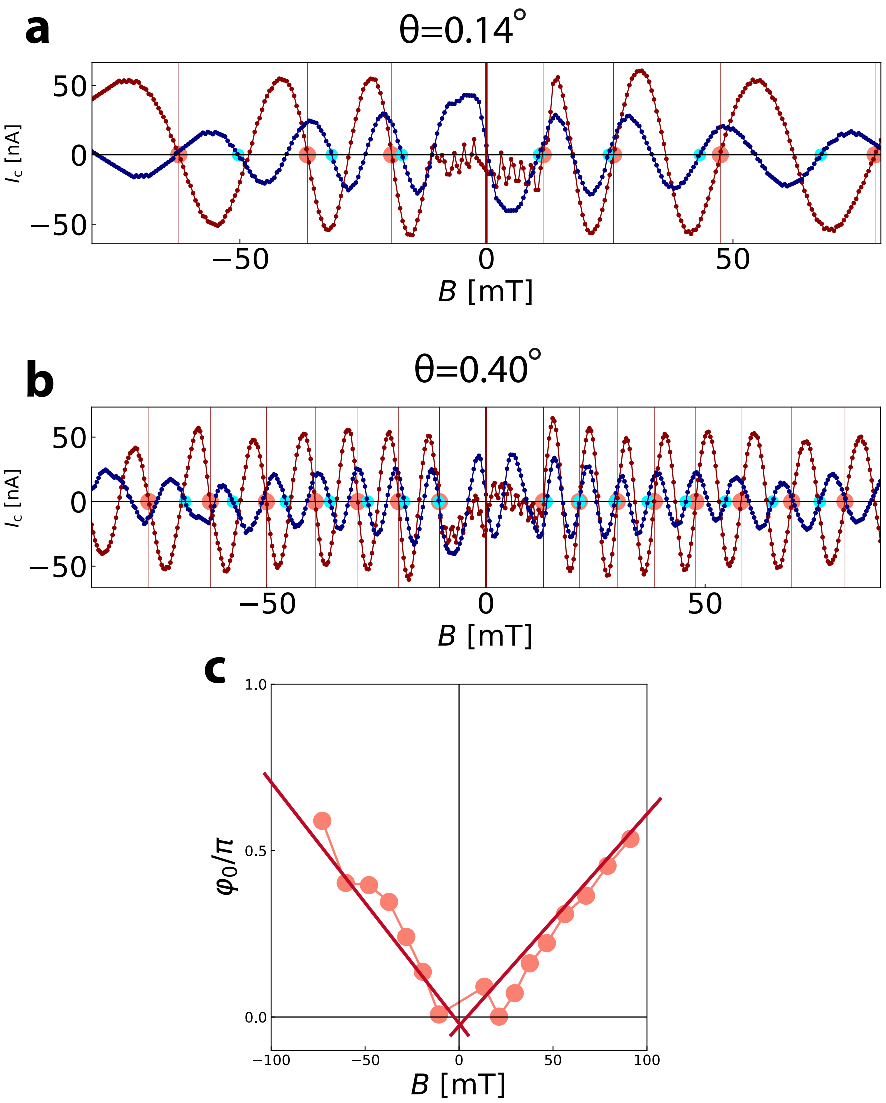

Supplementary Figure 7: **CPRs near zero magnetic field ab** On these CPRs taken at two different angles of the magnetic field with the sample, the nodes appearing every $2n\pi$ for $n = 0, 1, 2...$ are indicated by red (blue) dots for the reference (anomalous) JI. One can see that the two JIs are in-phase near zero magnetic field, i.e. the first two dots at positive and negative magnetic field, are on the top of each other. As the magnetic field increases, the blue dot shift to lower magnetic field, as a consequence of the anomalous phase shift. From these curves, the phase difference is extracted between each pair of dots(one red, one blue), thus, providing the phase difference between the two JIs as function of main field, shown panel **c**. This last plot shows that that the two JIs are in-phase at zero magnetic field and reach a dephasing approaching about $\pi/2$ for an in-plane magnetic field of $\simeq 80$ mT.

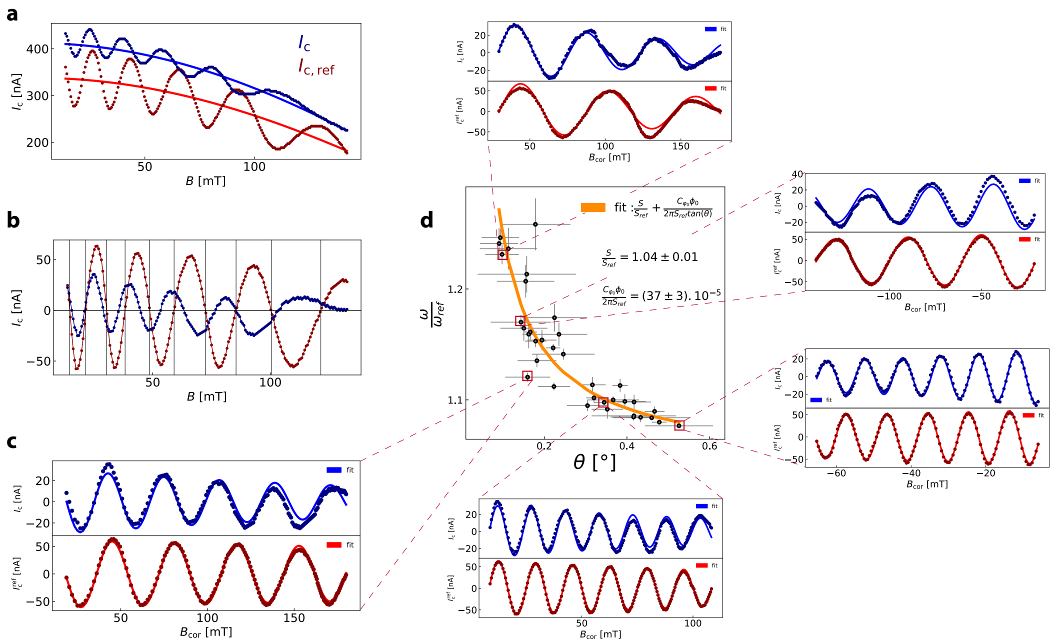

Supplementary Figure 8: **Comparison of the JIs frequencies as a function of the angle** θ**: second method.** **a** The critical current is shown as a function of magnetic field. The red and blue curves correspond to the reference and anomalous device respectively. The anomalous device shows a larger oscillation frequency than the reference device. As the critical current of the large junction decreases with the magnetic field, this leads to a decreasing background fitted for both devices and shown by the continuous lines. **b** Critical current as function of magnetic field. The background is subtracted to compare precisely the oscillation frequencies of the two devices. The zeros of critical current are not regularly spaced due to flux focusing. **c** Critical current of the anomalous (upper panel) and reference (lower panel) JI as a function of the corrected magnetic field $B_{\rm cor}$. The zeros of critical current are now regularly spaced. The frequencies for the reference and the anomalous devices are extracted from sinusoidal fit shown as continuous lines. **d** Ratio of the oscillation frequencies $\frac{\omega}{\omega_{\rm ref}}$ as a function of the angle θ. This ratio diverges as $\frac{1}{\theta}$ for small θ due to the anomalous phase generated by spin-orbit coupling.

Supplementary Tables

U$H(B_z, \varphi)$U†=H$(-B_z, \varphi)$	Broken by
$\sigma_x P_y$	B_y, α, V_y
$\sigma_y P_y$	B_x, V_y
$P_x P_y T$	B_x, B_y, α, V_x, V_y
$\sigma_z P_x P_y T$	V_x, V_y

Supplementary Table 1: **Symmetry operations U protecting** $H(B_z) = H(-B_z)$**, from Ref. [2].** The symmetry operations in the left column are broken by one of the parameters in the right column. These parameters include the in-plane magnetic fields B_x, B_y, the asymmetric disorder potentials V_x, V_y and the spin-orbit coefficient α. To observe a non symmetric Fraunhofer pattern, all symmetries in the first column need to be broken. One ingredient in the line of the second column is enough to break the corresponding symmetry. For example a non symmetric disorder along y, V_y, is enough to break all symmetries in the first column and induce a non symmetric Fraunhofer pattern, $I_c(B_z) \neq I_c(-B_z)$.

Supplementary Notes

Supplementary Note 1. Details on thin films growth. The samples were grown using a multi-chamber molecular beam epitaxy (MBE) setup at Institut des NanoSciences de Paris. High quality GaAs buffer layers were grown homoepitaxially on GaAs(111) in a Riber compact 21 MBE chamber. The substrates were then transferred under ultrahigh vacuum to a Riber 32 MBE chamber equipped with Bi and Se cells. Growth of Bi_2Se_3 epilayers was then carried out at $T = 300°C$ with a Se/Bi beam equivalent pressure ratio higher than 6.5. The growth temperature and Bi/Se flux ratio were fixed at optimal values within the growth parameters interval leading to high quality epilayers. Such values were determined by monitoring the crystalline quality in-situ by reflection high energy electron diffraction and ex-situ by x-ray diffraction, Supplementary Figure 1, and by post growth verification of the electronic structure (Dirac cone fingerprint in angle-resolved photoemission spectra[3]). The thickness of the films was checked by x-ray measurements, as shown Supplementary Figure 1. Following growth, the samples were capped with a Se protective layer.

Supplementary Note 2. A.C. Josephson effect. When irradiating a Josephson junction with a microwave signal, its phase oscillates with the frequency f of the applied signal. Standard junctions with a conventional 2π periodic CPR, $I_{\rm J} = I_{\rm c}\sin(\varphi)$, will display superfluid current steps, i.e. the Shapiro steps, at the voltage values $V_n = nhf/2e$.

The CPR of topological Majorana states has been predicted to be 4π periodic[4–6], which leads to fractional Shapiro steps. Fractional Shapiro steps have been observed in InSb nanowires driven in the topological regime by a magnetic field [7], in the 3D topological insulator HgTe [8], in the 2D

topological insulator HgTe quantum well [9] and in the Dirac semi-metal BiSb [10].

Bi_2Se_3 has been predicted to be a 3D topological insulator due to its strong spin-orbit interaction that leads to an inverted band structure [11]. The Dirac cone, which is a predicted characteristic of 3D topological insulators[11], has been observed by photoemission in the thin film used in this study [3].

To investigate the phase periodicity of the supercurrent we illuminate the junction with microwave. This leads to the resistance maps, shown in Fig. 2abcde, as a function of current and microwave power. Regular steps are observed in the IV curve, Fig. 2d, at voltage $V_n = nhf/2e$ where n is an integer and f is the microwave frequency. The behaviour is well captured by the resistively shunted junction (RSJ) model, Fig. 2f upper panel, with a conventional, 2π periodic, CPR. The absence of 4π periodicity is consistent with past experimental works [12–15]. Residual bulk conduction results in a supercurrent mediated predominantly by trivial states which are 2π periodic in phase.

RSJ model

The A.C. behaviour of a SNS Josephson junction can be captured by the RSJ model. The equivalent circuit of a SNS junction consists of a Josephson junction of critical current I_{c} in parallel with a resistor with a resistance R_{N}. The microwave signal is added in the model in the form of an ac current $I_{\mathrm{RF}} \sin(2\pi f t)$, with f the microwave frequency. The current in such a circuit is:

$$I = \frac{h}{2eR_{\mathrm{N}}}\frac{d\varphi}{dt} + I_{\mathrm{c}}\sin(\varphi) + I_{\mathrm{RF}}\sin(2\pi f t) \tag{1}$$

This equation is solved numerically for each pair of I and I_{RF}. The voltage V is found from the second Josephson relation by a time average $\langle\rangle_t$ of the phase solution derivative:

$$V = \frac{h}{2e}\langle\frac{d\varphi}{dt}\rangle_t \tag{2}$$

A 4π periodic contribution to the supercurrent can be added by using a CPR of the form $I_{\mathrm{J}} = I_{\mathrm{c}}(\frac{4}{5}\sin(\varphi) + \frac{1}{5}\sin(\varphi/2))$. The result is shown in the lower panel of Fig. 2f.

The RSJ model has been extended to understand the role of thermal effect which can mask the 4π periodic contribution to the supercurrent in Bi_2Se_3[16].

Supplementary Note 3. Periodicity of the Fraunhofer and Josephson interferometers. The first node of the Fraunhofer patterns measured on the junction described in the main text, Fig. 3, and the junctions described in Supplementary Figure 5 and Supplementary Figure 6abcd, are located at the value $B_0 \simeq 1.2$ mT. These two junctions have identical widths W=2 μm and electrode spacing $L = 150$ nm. The field position of the first node is expected at $B_0 = \frac{\phi_0}{W(L+2\lambda_z)}$.

For the aluminum electrodes, the effective penetration depth λ_{eff} is given by:

$$\lambda_{\text{eff}} = \lambda_{\text{L}}\sqrt{1+\frac{\xi}{\ell}} \quad (3)$$

where $\lambda_{\text{L}} = 16$ nm is the bulk London penetration depth of aluminum, ℓ is the mean free path and ξ is the coherence length[17] calculated from:

$$\xi = 0.36\sqrt{\frac{3\hbar v_{\text{F}}\ell}{2k_{\text{B}}T_{\text{c}}}} \quad (4)$$

From the measured critical temperature $T_{\text{c}} = 0.4$ K of the aluminum titanium bi-layer and mean free path of aluminum $\ell = \frac{\sigma m v_{\text{F}}}{ne^2} = 50$ nm, we find $\xi = 610$ nm and $\lambda_{\text{eff}} = 58$ nm.

For a magnetic field B_z perpendicular to a thin film, the penetration depth is given by[18]:

$$\lambda_z = \lambda_{\text{eff}} \coth(\frac{d}{\lambda_{\text{eff}}}) \quad (5)$$

For a film of thickness $d = 20$ nm, we find $\lambda_z = 175$ nm. With this value of London

penetration depth, we find $B_0 \simeq 2$ mT, which is about 2 times larger than the experimental value.

This discrepancy can be explained by flux-focusing which increases the magnetic field in the junction. A simple way to take this effect into account is described in Ref.[19]. When the electrode becomes superconducting, part of the magnetic flux lines that would penetrate the electrodes in the normal state are now expelled from the electrodes and focalized into the junction area. We can estimate the amount of flux focusing by considering the shortest distance a flux line has to be diverted to not pass through the superconducting electrode. This leads to an increase of the effective junction area from S to $S_{eff} = S + S_{focalized}$ where $S_{focalized} = 2 \times (W/2 - \lambda_z)^2$ as sketched Supplementary Figure 4. Taking into account this flux-focusing yields $B_0 \approx$ 0.9 mT, which is close to the experimental value.

In Supplementary Figure 5 and Supplementary Figure 6, abrupt jumps of the critical current magnitude are observed for $B_z \gtrsim 3$ mT. Such jumps are expected in presence of trapped vortices. Similar behavior has been observed in Josephson junctions fabricated with type II superconducting electrodes[20]. In our case, despite the modified London penetration depth and coherence length in our thin films, we have $\xi \approx 10\lambda_{\text{eff}}$, which implies that the aluminum remains of type I and no vortices are expected in the electrodes.

However, in hybrid Josephson junctions, the effective penetration depth in the semiconducting material is expected to be much larger than for the superconducting electrodes because the carrier density in the semiconductor is much smaller than in the electrodes. Thus, it is quite plausible that the hybrid heterostructure aluminum/semiconductor becomes a type II superconductor.

This probably explains the ubiquitous observation of flux-trapping effects in hybrids SNS junctions made from semiconducting materials[15,20].

As seen in Fig. 4, the period of the Josephson interferometers increase with in-plane magnetic field. The major ingredients for explaining the increasing period with magnetic field are first, the existence of flux-focusing and second, the increasing penetration depth with in-plane magnetic field.

As described above, flux-focusing leads to an increase of the effective junction area and so to a correspondingly short period. However, the increase in the penetration depth leads to a reduction in flux focusing as the magnetic lines that were diverted into the junction area are now penetrating the electrodes. This leads to a decrease of the effective area of the junction and so to an increase of the period. For example, in the Fraunhoffer pattern shown Fig. 5, the field position of the second critical current node appears at a larger magnetic field than expected from the field position of the first node. This effect has also been seen in other recent works[15,21]. This phenomena also occurs in the Josephson interferometers. As sketched Supplementary Figure 4, because of the finite width of the superconducting electrode making the interferometer, the effective area of the interferometer is larger than the inner area. Upon increasing the magnetic field, the increase in the penetration depth leads to a reduction of the effective area and so to an increase of the oscillation period of the interferometer, as shown Fig. 4, Fig. 5, Supplementary Figure 7 and Supplementary Figure 8.

The increase of the penetration depth with magnetic field could be due to the penetration of vortices in the electrodes or to a reduction in the amplitude of the superconducting order parameter

at the approach of the upper critical field. According to Ref. [22], the penetration depth increases as $\lambda_e ff \propto 1/\Delta(H) \rightarrow \infty$ for an in-plane magnetic field.

Finally, one also want to estimate the contribution from the circulating superfluid current, of maximum amplitude given by the critical current $I_c \simeq 1\ \mu$A, to the magnetic flux in the junction or JI.

For a Josephson junction, the screening of the applied magnetic field generated by the Josephson supercurrent is negligible when the characteristic size of the junction is smaller than the Josephson length:

$$\lambda_J = \left(\frac{\hbar}{2e\mu_0(L + 2\lambda_z)J_0} \right)^{1/2} \tag{6}$$

where J_0 is the current density. From this relation, one finds $\lambda_J \simeq 5\ \mu$m, which is larger than the characteristic lengths of the Josephson junctions where the large junctions have a size $2\ \mu$m$\times 150$ nm and the small junctions have a size 150 nm$\times 150$ nm. This implies negligible contribution of the Josephson supercurrent to the magnetic flux in the junctions.

For a JI, the actual maximum flux is given by $\phi = \phi_{applied} - L.I$, where L is the inductance of the JI loop. We estimate that the inductance of the JI loop is about $L \simeq 1.10^{-11}$ H. From this inductance, one finds $LI_c/\phi_0 = 10^{-2}$, showing that the superconducting current contribution to the total magnetic flux is negligible in comparison to the flux due to the applied magnetic field.

Supplementary Note 4. Simulation of the asymmetric Fraunhofer pattern A general relation for the critical current as a function of perpendicular B_z and in-plane magnetic field B_y is given by:

$$I_\mathrm{c}(B_z, B_y) = \max_{\varphi'} \int_{-W/2}^{W/2} \int_0^d j_0 \sin(\varphi' + \varphi_{xy}(y) + \varphi_{xz}(z) + \varphi_0(y))\mathrm{d}y\mathrm{d}z \quad (7)$$

where j_0 the critical current density, $d \simeq 20$ nm the thickness of the film, φ' an arbitrary global phase shift, $\varphi_{xy}(y) = \frac{2\pi(L+2\lambda_z)yB_z}{\phi_0}$ and $\varphi_{xz}(z) = \frac{2\pi(L+2\lambda_\mathrm{eff})zB_y}{\phi_0}$, are the magnetic fluxes produced in the junction by the magnetic field B_z and B_y respectively, where $L = 150$ nm is the distance between the superconducting electrodes.

Finally, the last phase argument $\varphi_0(y)$ in Supplementary Eq. 7 is the anomalous phase which is allowed to depend on y due to disorder[23]:

$$\varphi_0 = \frac{\tau m^{*2} E_\mathrm{Z}(\alpha L)^3}{3\hbar^6 D} \quad (8)$$

where $\tau = 0.13$ ps is the elastic scattering time, $D = 40$ cm^{2}s^{-1} is the diffusion constant, $m^* = 0.25\ m_e$ is the effective electron mass, $\alpha(y)$ is the spin-orbit coefficient that depends on disorder, $E_\mathrm{z} = \frac{1}{2}g\mu_\mathrm{B}B$ is the Zeeman energy with $g = 19.5$[24]. As shown by photoemission, the Rashba parameter depends on the thickness of the film[25]. Using Supplementary Eq. 8, one finds that a change of the Rashba coefficient by $\Delta\alpha = 0.01$ eV.Å leads to a phase jump of $\Delta\varphi_0 \simeq\ 0.3\pi$. This phase jump along the y-direction is sketched Supplementary Figure 6f and is sufficient to generate an asymmetric Fraunhofer pattern, as shown in Supplementary Figure 6e.

The mere existence of a large value for $\alpha = 0.38$ eVÅ does not lead to an asymmetric Fraunhofer pattern, in the absence of disorder, as the induced anomalous phase-shift φ_0 can always be compensated by the arbitrary phase φ'. In other words, because in Supplementary Eq.7 the critical current is obtained by maximizing over the arbitrary phase φ', a global change of φ_0 will be compensated by an equivalent change of the arbitrary phase φ'. Only anomalous phase jumps along the y direction can generate an asymmetric Fraunhofer pattern, in agreement with Supplementary Table 1, indicating that finite disorder V_y must be present for the asymmetry to be present.

AFM topographic images of our MBE films, Supplementary Figure 6gh, show that the film thickness changes by an amount ± 1 nm over a length $l_{\mathrm{d}} = 1$ μm. We model the variation in the spin-orbit coefficient by the function $\alpha(y) = \alpha_0 + \Delta\alpha \sin(2\pi y/l_{\mathrm{d}})$ where $\Delta\alpha = 0.01$ eVÅ. The resulting Fraunhofer pattern is shown in Supplementary Figure 6hk, displaying good agreement with the experimental data.

Supplementary Note 5. Comparison of the JIs frequencies as a function of the angle θ (second method). In addition to the method described in the main text for extracting the frequency ratio $\frac{\omega}{\omega_{\mathrm{ref}}}$ between the two interferometers, we present here a second method based on a rescaling of the applied magnetic field. To compare the frequencies of the two interferometers we first remove the critical current background. This is done by fitting the $I_{\mathrm{c}}(B)$ curve of the two devices with a parabola, Supplementary Figure 8a, which is removed from the experimental data and shown Supplementary Figure 8b.

The oscillation frequency of the reference interferometer should not vary with the amplitude

of the magnetic field applied with a small angle θ with the plane of the sample. However, because of flux focusing effects, this frequency is observed to change with the the magnetic field, Supplementary Figure 8b.

To correct for this flux focusing effect, we rescale the magnetic field, giving the corrected magnetic field scale B_{cor}, such that the reference signal becomes periodic as shown in Supplementary Figure 8c. This same corrected scale is applied to the anomalous device. In this corrected field scale, the frequency ratio $\frac{\omega}{\omega_{\mathrm{ref}}}$ is extracted and plotted Supplementary Figure 8d. At large θ, this ratio is equal to the ratio of areas $S/S_{\mathrm{ref}} \simeq 1$, however, for small θ, this ratio increases as $1/\tan(\theta)$, indicating the presence of an anomalous phase shift $\varphi_0 = C_{\varphi_0} B$.

In Supplementary Figure 8b and in the main text, the angle θ is determined from the last oscillation period ΔB of the reference device by the relation $\theta = \arcsin(\frac{\phi_0}{S\Delta B})$. The error on the determination of the angle $\delta\theta$ is due to the error $\delta(\Delta B)$ which is estimated as the difference between the last oscillation period and the before last oscillation period. The error on the angle is given by the relation $\delta\theta = \frac{\phi_0 \delta(\Delta B)}{S\Delta B^2 \sqrt{1-\left(\frac{\phi_0}{S\Delta B}\right)^2}}$.

Supplementary References

1. Hyde, G. R. *et al.* Shubnikov-de haas effects in Bi2Se3 with high carrier concentrations. *Solid State Commun.* **13**, 257–263 (1973).

2. Rasmussen, A. *et al.* Effects of spin-orbit coupling and spatial symmetries on the josephson current in SNS junctions. *Phys. Rev. B Condens. Matter* **93**, 155406 (2016).

3. Vidal, F. *et al.* Photon energy dependence of circular dichroism in angle-resolved photoemission spectroscopy of Bi_2Se_3 dirac states. *Phys. Rev. B Condens. Matter* **88**, 241410 (2013).

4. Snelder, M., Veldhorst, M., Golubov, A. A. & Brinkman, A. Andreev bound states and current-phase relations in three-dimensional topological insulators. *Phys. Rev. B Condens. Matter* **87**, 104507 (2013).

5. Yu Kitaev, A. Unpaired majorana fermions in quantum wires. *Phys.-Usp.* **44**, 131 (2007).

6. Fu, L. & Kane, C. L. Josephson current and noise at a superconductor/quantum-spin-hall-insulator/superconductor junction. *Phys. Rev. B Condens. Matter* **79**, 161408 (2009).

7. Rokhinson, L. P., Liu, X. & Furdyna, J. K. The fractional a.c. josephson effect in a semiconductor–superconductor nanowire as a signature of majorana particles. *Nat. Phys.* **8**, 795 (2012).

8. Wiedenmann, J. *et al.* 4π-periodic josephson supercurrent in HgTe-based topological josephson junctions. *Nat. Commun.* **7**, 10303 (2016).

9. Bocquillon, E. *et al.* Gapless andreev bound states in the quantum spin hall insulator HgTe. *Nat. Nanotechnol.* **12**, 137–143 (2017).

10. Li, C. *et al.* 4π-periodic andreev bound states in a dirac semimetal. *Nat. Mater.* **17**, 875–880 (2018).

11. Zhang, H. *et al.* Topological insulators in Bi2Se3, Bi2Te3 and Sb2Te3 with a single dirac cone on the surface. *Nat. Phys.* **5**, 438 (2009).

12. Kurter, C., Finck, A. D. K., Hor, Y. S. & Van Harlingen, D. J. Evidence for an anomalous current-phase relation in topological insulator josephson junctions. *Nat. Commun.* **6**, 7130 (2015).

13. Galletti, L. *et al.* Influence of topological edge states on the properties of $AlBi_2Se_3Al$ hybrid josephson devices. *Phys. Rev. B Condens. Matter* **89**, 134512 (2014).

14. Cho, S. *et al.* Symmetry protected josephson supercurrents in three-dimensional topological insulators. *Nat. Commun.* **4**, 1689 (2013).

15. Williams, J. R. *et al.* Unconventional josephson effect in hybrid superconductor-topological insulator devices. *Phys. Rev. Lett.* **109**, 056803 (2012).

16. Le Calvez, K. *et al.* Joule overheating poisons the fractional ac josephson effect in topological josephson junctions (2018). Preprint at http://arxiv.org/abs/1803.07674.

17. Larkin, A. *Theory of ∞uctuations in superconductors*.

18. Gubin, A. I., Il'in, K. S., Vitusevich, S. A., Siegel, M. & Klein, N. Dependence of magnetic penetration depth on the thickness of superconducting nb thin films. *Phys. Rev. B Condens. Matter* **72**, 064503 (2005).

19. Molenaar, C. G., Leusink, D. P., Wang, X. L. & Brinkman, A. Geometric dependence of Nb-Bi2Te3-Nb topological josephson junction transport parameters. *Supercond. Sci. Technol.* **27**, 104003 (2014).

20. Kim, B.-K. *et al.* Strong superconducting proximity effects in PbS semiconductor nanowires. *ACS Nano* **11**, 221–226 (2017).

21. Suominen, H. J. *et al.* Anomalous fraunhofer interference in epitaxial superconductor-semiconductor josephson junctions. *Phys. Rev. B Condens. Matter* **95**, 035307 (2017).

22. Tinkham, M. *Introduction to superconductivity - 2nd ed* (New York : McGraw Hill, 1996).

23. Bergeret, F. S. & Tokatly, I. V. Theory of diffusive φ 0 josephson junctions in the presence of spin-orbit coupling. *EPL* **110**, 57005 (2015).

24. Wolos, A. *et al.* g -factors of conduction electrons and holes in B i 2 S e 3 three-dimensional topological insulator. *Phys. Rev. B Condens. Matter* **93**, 3023 (2016).

25. Zhang, Y. *et al.* Crossover of the three-dimensional topological insulator Bi2Se3 to the two-dimensional limit. *Nat. Phys.* **6**, 584 (2010).
